# Ergonomic working posture in simulated dental clinical conditions: effect of magnification on the operator’s experience

**DOI:** 10.7717/peerj.11168

**Published:** 2021-04-08

**Authors:** Danielle Wajngarten, Júlia Margato Pazos, Patricia Petromilli Nordi Sasso Garcia

**Affiliations:** Social Dentistry, São Paulo State University (Unesp), School of Dentistry, Araraquara, São Paulo, Araraquara, São Paulo, Brasil

**Keywords:** Dental education, Professional practice, Magnification

## Abstract

**Objectives:**

This study observed the effect of different magnification systems on working posture and neck angulation during cavity preparation procedures according to operator’s experience.

**Methods:**

This was a laboratory study. The response variables were the neck angulation and the working posture adopted during Class I cavity preparations (*N* = 640) that were performed under four conditions (unaided visualization, simple loupe, Galilean loupe and Keplerian loupe). Working postures were recorded and evaluated by the Compliance Assessment of Dental Ergonomic Posture Requirements (CADEP). The two-factor ANOVA and Games-Howell post-hoc test were performed (*α* = 0.05).

**Results:**

For all treated teeth it was observed higher posture scores and lowest neck angulations while using the Galilean and Keplerian loupes (*p* < 0.01). No correlations were found between operator’s experience and working posture (*p* = 0.71–0.88).

**Conclusion:**

It can be concluded that Galilean and Keplerian loupes helped operators to maintain an ergonomic posture and lower neck angulations for all teeth and the operator’s experience provided better ergonomic posture for the mandibular teeth.

## Introduction

Dental surgeons must work in a small operating field under dark or low-light conditions. As a result, their visualization of and access to oral structures are compromised (Presoto & Garcia, 2016; Presoto, Wajngarten & Garcia, 2016). Because this visibility is of extreme importance for dental treatment (Perrin, Jacky & Hotz, 2000), professionals often instinctively move closer to patients in an attempt to improve visualization of the anatomical structures of the oral cavity (Graça, Araújo & SIlva, 2006; Keinan, Nuni & Slutzky-Goldberg, 2009; De Jesus Júnior & Campos, 2014). Because of this focus on the procedure, both dental students and professionals find themselves disregarding their working posture.

This behavior compromises the position of the head, torso, and shoulders (De Jesus Júnior & Campos, 2014; Van As, 2014) and may lead to the development of musculoskeletal disorders (Oberg & Oberg, 1993; Garcia, Polli & Campos, 2013; Corrocher et al., 2014; Gupta et al., 2014; Sio et al., 2018). To better meet the visual demands required in dental treatments without compromising dental professionals’ musculoskeletal health, magnifying lenses have been recommended with increasing frequency (Sio et al., 2018; Christensen, 2003; Maillet et al., 2008; Narula et al., 2015).

In addition to ergonomic benefits, magnification may also improve fine motor skills, diagnostic abilities, and the quality of surgical procedures (Maillet et al., 2008; Narula et al., 2015; Bowers et al., 2010; Eichenberger et al., 2015); however, scientific evidence to support these benefits is scarce (Christensen, 2003; Wajngarten & Garcia, 2016; Branson et al., 2004).

The implementation of magnification devices is useful as early as the pre-clinical phase of training because musculoskeletal disorders may develop as early as the training phase of their careers (Corrocher et al., 2014; Garcia et al., 2012). It may even lead to students withdrawing from their programs (Plessas & Bernardes Delgado, 2018). Even so, the use of these devices may be a challenge for students with limited procedural experience (Carpentier et al., 2019).

For these reasons, the objective of this study was to observe the effect of different magnification systems on working posture and neck angulation during procedures involving cavity preparation on artificial teeth by operators with and without experience in restorative dentistry and dentistry-related ergonomics.

## Materials and Methods

This study was approved by the Research Ethics Committee of the School of Dentistry of São Paulo State University (UNESP), Araraquara Campus (CAAE Registry No. 4753816.9.0000.5416). Participants agreed with the written consent form and participated in this research study voluntarily.

The experimental design of this study followed the design proposed by Pazos et al. (2020): it was performed a laboratory study. The response variables were angulation of the neck and the working posture adopted during cavity preparation procedures in restorative dentistry. The working posture adopted while performing the simulated clinical procedures in dental mannequin was evaluated by CADEP method (Garcia, Wajngarten & Campos, 2018). The independent variables were the magnification system under four conditions (unaided visualization, the use of a simple loupe, the use of a Galilean loupe, and the use of a Keplerian loupe) and operator’s experience under two conditions (inexperienced or experienced operators). The inexperienced operator was an undergraduate dental student and the experienced operators was a graduate student. The sample unit was the prepared teeth and the minimal sample size was determined using data from a pilot study, a power of 80% and a significance level of 5%. This resulted in 20 teeth in each experimental condition. Tooth and loupes were randomized so that 20 cavity preparations of each tooth (16, 26, 36, 46) were carried out with each of the magnifying loupes (*N* = 320) and operator (*N* = 640).

The inclusion criteria for the participation were the absence of prior health problems that could possibly affect the psychomotor ability and visual acuity, as well as being in the dentistry field.

### Magnification devices

In this study the magnification devices used were: simple loupe 3.5x magnification (BioArt - Brazil), Galilean device 3.5x magnification (Ymarda Optical Instrument Factory, Nanjing - China) and Keplerian device 4.0x magnification (Ymarda Optical Instrument Factory, Nanjing - China).

### Cavity preparations

Class I cavity preparations were performed according Pazos et al. (2020): Class I for composite resin on tooth numbers 16 (right maxillary first molar), 26 (left maxillary first molar), 36 (left mandibular first molar), and 46 (right mandibular first molar).

The cavity preparation procedures were performed following the technique taught in the Restorative Dentistry I course of the School of Dentistry of São Paulo State University (UNESP), Araraquara Campus, according to Baratieri & Monteiro Jr (2015). A diamond bur (Kg Sorensen Model 1014) was used on low rotation and the preparation needed to exhibit rounded internal line angles to support the force of mastication and a depth/width corresponding to 1 to 1.5 burs.

A MOM-brand dental mannequin (Marília, São Paulo), which has artificial resin teeth specific for cavity preparation at the pre-clinical level, was used in the procedures. As the teeth were prepared, they were removed and replaced by intact resin teeth so that new preparations could be performed. The mannequins were placed in dental chairs to simulate a clinical setting.

### Working postures

Working postures were recorded using digital video cameras. The cameras (GoPro –Hero 4) and tripods were positioned in such a way that all of the body parts and equipment under assessment could be adequately filmed, regardless of the tooth being prepared (Pazos et al., 2020).

### Posture assessment

A calibrated researcher (ρ = 1.0), different from the one who made the procedures, evaluated the working posture based on the video recordings that was taking during the entire dental procedure. The posture most frequently adopted by the operator was evaluated using a modified version of the Compliance Assessment of Dental Ergonomic Posture Requirements (CADEP) proposed by Garcia, Wajngarten & Campos, 2018. This instrument considers the items presented in Table 1.

**Table 1:**
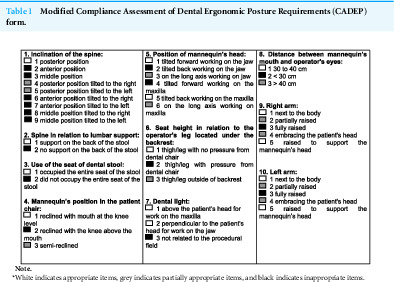
Modified Compliance Assessment of Dental Ergonomic Posture Requirements (CADEP) form.

**Note.**

* White indicates appropriate items, grey indicates partially appropriate items, and black indicates inappropriate items.

The items presented in Table 1 were evaluated and classified as ergonomically appropriate, partially ergonomically appropriate, and ergonomically inappropriate. Each item received a score based on its classification: one point was awarded for each appropriate item (items which were in accordance with basic requirements for ergonomic posture), zero points were awarded for inappropriate items (when the requirements were not met), and half a point was awarded for partially appropriate items, or when the item evaluated was not completely correct (Garcia, Wajngarten & Campos, 2018). After each item received a score, the points were added up, with a maximum possible score of ten points.

After each item received a score, the points were added up, with a maximum possible score of ten points.

### Evaluating angulation

The position of the camera/tripod system allowed for a lateral view of the operators, thus enabling an evaluation of the angulation of the neck at a neutral posture.

Angulation was measured following the method used by Wajngarten & Garcia (2019): a single trained researcher (ρ = 0.88) using a local posture evaluation software known as *Software para Avaliação Postural*, version 0.69 (Laboratory for Biomechanics and Motor Control Federal University of ABC [UFABC], São Bernardo do Campo, São Paulo State, Brazil) performed the angular evaluation.

### Statistical analysis

The data on the dependent variables (working posture and neck angulation) was analyzed independently for the different teeth (numbers 16, 26, 36, and 46) in an attempt to determine any differences between the loupes and the operators relative to the dental arch on which each tooth under study was located.

After meeting the assumptions of normality (Sk = 0.16–2.08; Ku = 0.14–4.41), the two-factor analysis of variance (ANOVA) was performed with Welch’s *t*-test and the Games-Howell post-hoc test (α = 0.05).

## Results

The mean, standard deviation, and summary of the ANOVA of the final score for the working postures adopted during the cavity preparation procedures on tooth numbers 16, 26, 36, and 46 and organized by operator’s experience and magnification device can be found in Table 2.

**Table 2:** Mean, standard deviation, and summary of the ANOVA of the final score for the working postures used during the cavity preparation procedures on tooth numbers 16, 26, 36, and 46 and organized by operator’s experience and magnification device.

Tooth number	Operator+	Magnification Device															
		Naked eye		Simple loupe		Galilean loupe		Keplerian loupe		Source of Variation++	SS	df	MS	F	*p*	*η*_p_^2^	*π*
16	1	8.92	±0.65	8.60	±0.38	9.72	±0.38	9.85	±0.28	A	0.014	1	0.014	0.072	0.789	0.000	0.058
	2	8.87	±0.51	8.57	±0.44	9.80	±0.38	9.77	±0.41	B	45.580	3	15.193	77.659	<0.001	0.605	1.000
										A*B	0.130	3	0.043	0.221	0.882	0.004	0.091
26	1	8.82	±0.61	8.57	±0.61	9.75	±0.38	9.80	±0.38	A	0.039	1	0.039	0.135	0.714	0.001	0.065
	2	8.92	±0.89	8.47	±0.57	9.90	±0.21	9.77	±0.34	B	51.430	3	17.143	59.038	<0.001	0.538	1.000
										A*B	0.392	3	0.131	0.450	0.718	0.009	0.139
36	1	8.47	±0.73	8.37	±0.53	9.45	±0.56	9.70	±0.38	A	6.602	1	6.602	20.673	<0.001	0.120	0.995
	2	7.97	±0.68	7.92	±0.52	9.17	±0.57	9.30	±0.47	B	60.230	3	20.077	62.872	<0.001	0.554	1.000
										A*B	0.280	3	0.093	0.292	0.831	0.006	0.105
46	1	8.52	±0.85	8.50	±0.49	9.65	±0.56	9.72	±0.41	A	6.806	1	6.806	16.995	<0.001	0.101	0.984
	2	8.02	±0.73	8.27	±0.68	9.17	±0.61	9.27	±0.62	B	51.031	3	17.010	42.474	<0.001	0.456	1.000
										A*B	0.481	3	0.160	0.401	0.753	0.008	0.128

**Notes.**

+1= experienced operator; 2= inexperienced operator.

++A= operator’s experience; B= magnification device.

Higher posture scores were observed while using the the Galilean and Keplerian loupes. No correlations were found between operator’s experience and working posture score for the upper arch. A significant correlation was found between magnification device and working posture score for both arches.

The mean, standard deviation, and summary of the ANOVA of the neck angulation observed during the cavity preparation procedures on tooth numbers 16, 26, 36, and 46 and organized by operator’s experience and magnification device can be found in Table 3.

**Table 3:** Mean, standard deviation, and summary of the ANOVA of the final score for the working postures used during the cavity preparation procedures on tooth numbers 16, 26, 36, and 46 and organized by operator’s experience and magnification device.

Tooth number	Operator+	Magnification Device															
		Naked eye		Simple loupe		Galilean loupe		Keplerian loupe		Source of Variation++	SS	df	MS	F	*p*	*η*_p_^2^	*π*
16	1	44.67	±8.05	45.24	±5.78	32.48	±6.40	29.15	±6.63	A	27.970	1	27.970	0.630	0.430	0.004	0.12
	2	42.96	±7.64	41.84	±6.28	32.91	±7.07	30.48	±5.08	B	6340.780	3	2113.590	47.350	<0.001	0.480	1.00
										A*B	136.240	3	45.410	1.020	0.390	0.020	0.27
26	1	45.24	±5.78	46.04	±7.40	31.95	±8.23	28.07	±8.20	A	325.470	1	325.470	0.510	0.480	0.003	0.11
	2	41.52	±6.55	41.52	±6.55	46.54	±8.28	31.82	±7.67	B	5017.470	3	1672.490	25.190	<0.001	0.50	1.00
										A*B	2185.700	3	728.570	1.140	0.330	0.02	0.30
36	1	45.30	±9.77	45.72	±7.62	29.98	±9.40	38.65	±5.16	A	1800.290	1	1800.290	0.510	0.050	0.003	0.11
	2	39.20	±7.25	39.24	±9.33	27.61	±5.89	33.20	±9.13	B	5701.250	3	1900.420	4.580	<0.010	0.840	0.88
										A*B	458.880	3	152.960	0.370	0.770	0.007	1.12
46	1	46.79	±9.50	44.64	±8.18	30.60	±9.24	24.73	±8.57	A	59.170	1	59.170	0.830	0.360	0.005	0.148
	2	41.84	±8.58	41.24	±8.19	31.41	±5.90	27.41	±7.77	B	9631.220	3	3210.410	44.890	<0.010	0.470	1.000
										A*B	379.910	3	126.640	1.770	0.150	0.034	0.455

**Notes.**

+1= experienced operator; 2= inexperienced operator.

++A= operator’s experience; B= magnification device.

The Galilean and Keplerian loupes allowed the lowest angulations of the neck. No correlations were found between operator’s experience and angulation of the neck, regardless of the tooth being treated. A significant correlation was found between the magnification device and angulation of the neck, regardless of the tooth being treated.

The absolute and relative frequencies of the CADEP items scored as appropriate, partially appropriate, and inappropriate are presented in Table 4.

**Table 4:** Absolute and relative frequencies of the items evaluated on the CADEP and organized by score category.

Item	Operator+			
	1		2	
**Tilting of the spine**	**n**	**%**	**n**	**%**
Appropriate	179	55.9	260	81.3
Partially appropriate	141	44.1	60	18.8
Inappropriate	–		–	–
**Spine in relation to lumbar support:**				
Appropriate	320	100	320	100
Inappropriate	–		–	
**Use of the seat of dental stool:**				
Appropriate	320	100	320	100
Inappropriate	–		–	
**Mannequin’s position in the patient chair**				
Appropriate	319	99.7	165	51.6
Partially appropriate	–		155	48.4
Inappropriate	1	0.3	–	–
**Position of mannequin’s head**				
Appropriate	319	99.7	153	47.8
Partially appropriate	1	0.3	167	52.2
Inappropriate	–	–	–	–
**Seat height in relation to the operator’s leg located under the backrest**				
Appropriate	312	97.5	315	98.4
Partially appropriate	–		–	–
Inappropriate	8	2.5	5	1.6
**Dental light**				
Appropriate	309	96.6	278	86.9
Inappropriate	11	3.4	42	13.1
**Distance between mannequin’s mouth and operator’s eyes**				
Appropriate	171	53.4	156	48.8
Partially appropriate	2	0.6	–	–
Inappropriate	149	46.6	164	51.3
**Right arm**				
Appropriate	300	93.8	292	91.3
Partially appropriate	20	6.3	–	–
Inappropriate	–		164	51.3
**Left arm**				
Appropriate	314	98.1	214	66.9
Partially appropriate	5	1.6	106	33.1
Inappropriate	1	0.3	–	–

**Notes.**

+1= experienced operator.

2= inexperienced operator.

**Figure 1:**
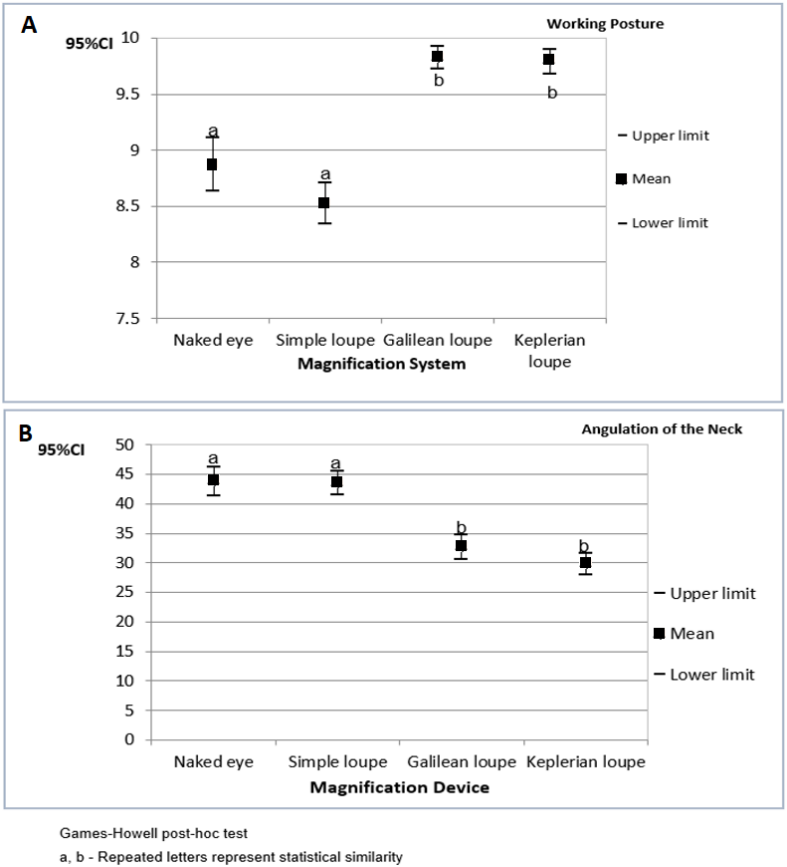
The 95% confidence interval (CI_95%_) for the operators’ working posture scores (A) and angulations of the neck (B) during cavity preparations performed on tooth number 16 (results organized by magnification device).

Overall, most of the items were classified as appropriate. Figure 1 presents the confidence interval for the operators’ working posture scores and angulations of the neck during cavity preparations performed on tooth number 16. The results are organized by magnification device.

The highest working posture scores and the lowest angulations of the neck were found when the operators used Galilean and Keplerian loupes.

**Figure 2:**
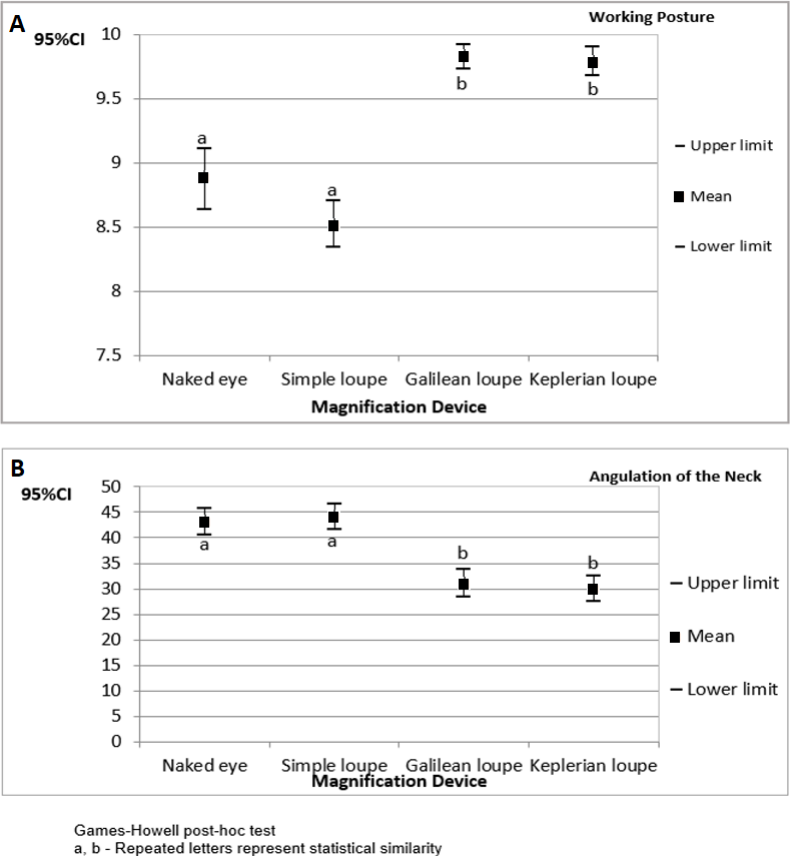
The 95% confidence interval (CI_95%_) for the operators’ working posture scores (A) and angulations of the neck (B) during cavity preparations performed on tooth number 26 (results organized by magnification device).

Figure 2 presents the confidence interval for the operators’ working posture scores and angulations of the neck during cavity preparations performed on tooth number 26. The results are organized by magnification device.

Again, the highest working posture scores and the lowest angulations of the neck were found when the operators used Galilean and Keplerian loupes on this tooth.

In the procedures performed on the mandibular teeth (numbers 36 and 46), working posture was found to be significantly associated with both the magnification device (F_(36)_ = 62.872, p_(36)_ < 0.001, F_(46)_ = 42.474, p_(46)_ < 0.001) and the operator’s experience (F_(36)_ = 20.673, p_(36)_ < 0.001, F_(46)_ = 16.995, p_(46)_ < 0.001). Angulation of the neck, meanwhile, was found to be significantly associated only with the magnification device (F_36_) = 4.580, p_(36)_ < 0.001, F_(46)_ = 44.890, p_(46)_ < 0.001).

**Figure 3:**
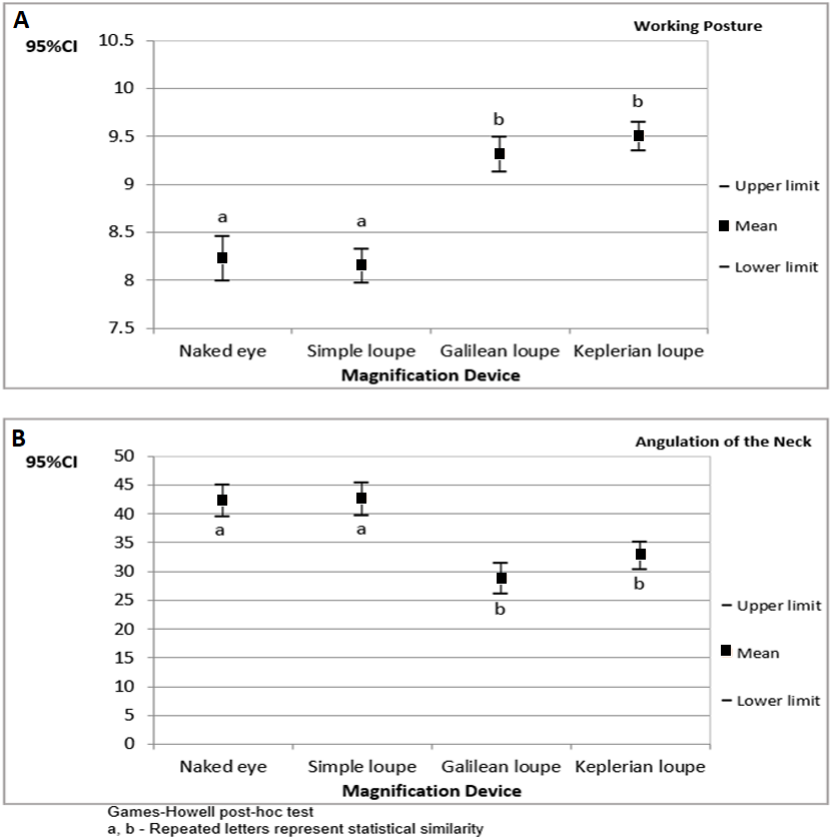
The 95% confidence interval (CI_95%_) for the operators’ working posture scores (A) and angulations of the neck (B) during cavity preparations performed on tooth number 36 (results organized by magnification device).

Figure 3 presents the confidence interval for the operators’ working posture scores and angulations of the neck during cavity preparations performed on tooth number 36. The results are organized by magnification device.

The highest working posture scores and the lowest angulations of the neck were found when the operators used Galilean and Keplerian loupes on this tooth.

**Figure 4:**
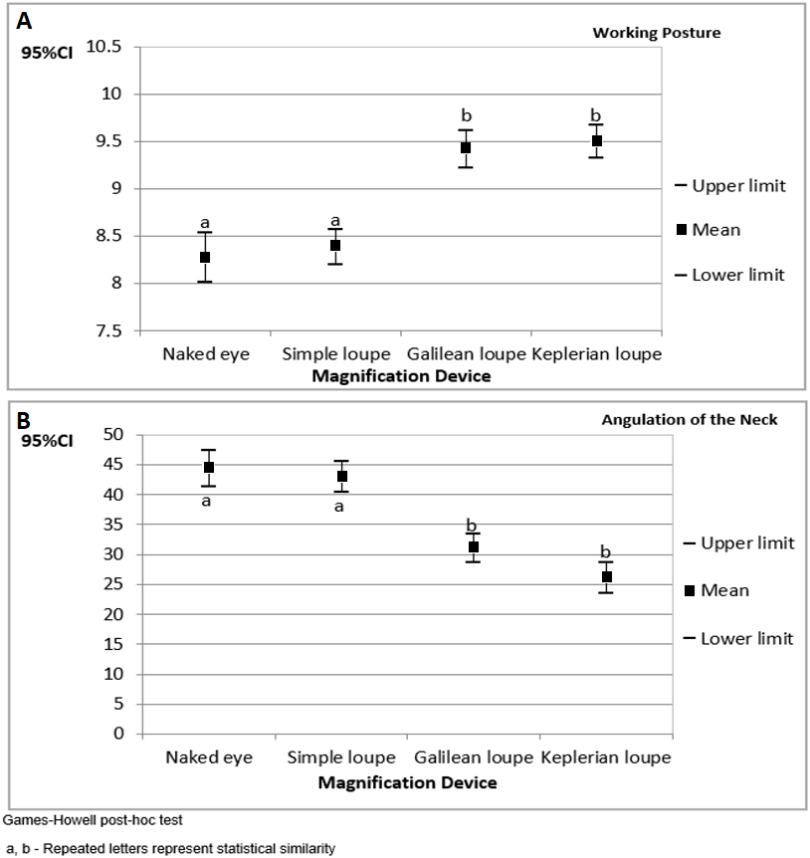
The 95% confidence interval (CI_95%_) for the operators’ working posture scores (A) and angulations of the neck (B) during cavity preparations performed on tooth number 46 (results organized by magnification device).

Figure 4 presents the confidence interval for the operators’ working posture scores and angulations of the neck during cavity preparations performed on tooth number 46. The results are organized by magnification device.

The highest working posture scores and the lowest angulations of the neck were found when the operators used Galilean and Keplerian loupes on this tooth.

**Figure 5:**
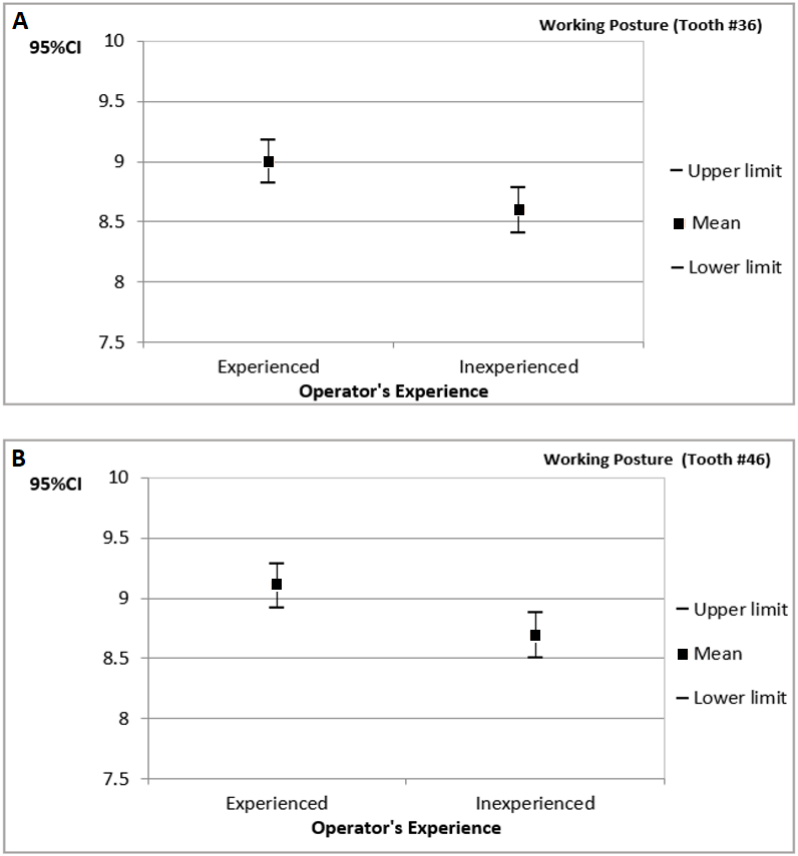
The 95% confidence interval (CI_95%_) for the operators’ working posture scores during cavity preparations performed on tooth numbers 36 (A) and 46 (B) (results organized by operator’s experience).

Figure 5 presents the confidence interval for the operators’ working posture scores during cavity preparations performed on tooth numbers 36 and 46. The results are organized by the operator’s experience.

The highest posture scores were found when cavity preparations were performed on tooth numbers 36 and 46 by the experienced operator.

## Discussion

The objective of this study was to observe the effects of different magnification devices on working posture and angulation of the neck of experienced and inexperienced dentistry students during pre-clinical class I cavity preparation procedures.

Overall, the Galilean and Keplerian loupes were found to be associated with the best working postures; the posture scores associated with these loupes were higher than those associated with the naked eye and the simple loupe.

Branson et al. (2004) and Maillet et al. (2008) evaluated students’ working posture during periodontal procedures (drilling and dental scaling, respectively) with and without magnification and found positive effects on individuals’ working postures when the procedures were performed under magnification. Dable et al. (2014) evaluated the risk of musculoskeletal disorders among students who used or did not use magnification devices and conventional or ergonomic seats. They found that students who worked sitting on a conventional seat and without magnification experienced a higher risk of musculoskeletal problems.

In our study, we found that the inexperienced operator received a lower working posture score when performing cavity preparation on the mandibular teeth. Garcia et al. (2012) and Corrocher et al. (2014) also evaluated inexperienced operators; however, they found no correlations between the region of the mouth being treated and postures that put operators at a higher risk of musculoskeletal disorders.

The descriptive statistical analysis of the CADEP items evaluated herein (Table 3) allows us to suppose that the lower scores received by the inexperienced operator during the cavity preparations performed on the mandibular teeth were largely associated with the position of the dental chair and of the mannequin’s head, as well as with the posture of the operator’s left (and dominant) arm. The inexperienced operator tended to place the dental chair in an only partially appropriate position (semi-reclined). When an operator is working on the lower dental arch and places the dental chair in a semi-reclined position, the view of the occlusal surface of the posterior teeth may be limited when the patient’s head is facing forward. Thus, it is likely that the inexperienced operator positioned the mannequin’s head in an only partially appropriate position (along the axis of the dental chair) more frequently as a strategy to compensate for this difficulty.

Unlike the operator’s working posture, the angulation of the neck was not affected by the operator’s experience working on the lower dental arch. The magnification devices were likely to have compensated for the only partially appropriate position of the dental chair and helped to prevent this position from affecting the angulation of the operator’s neck.

Dentistry students have difficulty positioning patients in general, particularly when these students are transitioning between pre-clinical and clinical activities. Students in this stage of their training are typically insecure about working with patients (Presoto, Wajngarten & Garcia, 2016) and they are often concerned about lowering the chair too far and making patients uncomfortable. These concerns have a negative effect on students’ working postures. At the time of this study, the inexperienced student was at precisely this transitional moment in the program, a factor which reinforces this explanation of the results.

When neck angulation was considered, it was found that the binocular loupes (Galilean and Keplerian) resulted in less angulation than the monocular loupe and the naked eye, regardless of the operator’s experience or the quadrant in which the procedure was performed. These results demonstrate that the optic complexity of the binocular loupes provides an adequate working distance (Perrin et al., 2016), and that these lenses therefore allow for lower angulation of the neck when obtaining the ideal angle for visualization. Branson et al. (2018) evaluated Galilean loupes using motion capture technology and found that the dentists studied employed less head flexion while using magnification devices.

After the fabrication of a large number of cavity preparations using a Galilean device (*n* = 80) and a Keplerian device (*n* = 80), the operators in our study were able to perceive differences between the devices. The operators reported that the Galilean loupe offered easier adaptation, greater comfort and adjustment options, better visualization of the procedural field, and increased clarity. When compared to the Galilean loupe, the Keplerian loupe was found to offer less clarity and a reduced visual field; the Keplerian loupe also resulted in a loss of focus when the operators made small movements.

It is important to note that the use of magnification devices alone is not necessarily sufficient to establish an ideal working posture. Only when they are combined with the teaching of ergonomic principles can magnification devices improve the occupational health of future dentistry professionals.

## Conclusion

It can be concluded that the Galilean and Keplerian magnification lenses helped the dental students of this study to maintain an ergonomic posture and lower neck angulations for all teeth and the operator’s experience provided better ergonomic posture for the mandibular teeth. So, these magnification devices can be used successfully even during the preclinical phase of dentistry training. Due to its ease of adjustment, its lower cost, and its adaptability, the Galilean could be recommended for new users. Therefore, magnification devices should therefore be implemented in dentistry training programs as early as the pre-clinical activities.

##  Supplemental Information

10.7717/peerj.11168/supp-1 Supplemental Information 1 Raw data used to conduct statistical analysis

## Additional Information and Declarations

### Data Availability

The following information was supplied regarding data availability: Raw data, including CADEP scores and neck angulation of all cavity preparations made by the experienced and inexperienced operators, and the magnification used, are available as a Supplemental File.
